# Trauma or Drama: A Predictive Processing Perspective on the Continuum of Stress

**DOI:** 10.3389/fpsyg.2020.01248

**Published:** 2020-06-30

**Authors:** Valery Krupnik

**Affiliations:** Department of Mental Health, Naval Hospital Camp Pendleton, Camp Pendleton, CA, United States

**Keywords:** trauma, allostasis, stress response, predictive processing, precision weighting

## Abstract

The notion of psychological trauma has been liberally used both in clinical literature and general discourse. However, no consensus exists on its exact meaning and definition. Whereas traditionally trauma has been mostly associated with criterion A of acute and posttraumatic stress disorders (PTSDs) as defined in the *Diagnostic and Statistical Manual of Mental Disorders*, many researchers find this definition too constraining and not accounting for the complexity and many aspects of trauma. This touched off a quest for a broader more accommodating trauma concept, and a dimensional view of trauma with PTSD as its extreme manifestation has been suggested. The dimensional view also has its detractors arguing that “conceptual bracket creep” may undermine the category’s utility. Both categorical and dimensional views mostly rely on trauma’s clinical phenomenology and lack a unified theoretical basis. In an attempt to reconcile this contradiction, a hybrid categorical–dimensional model of trauma based on the general theory of stress has been recently proposed (Krupnik, 2019). Herein, I explore the categorical boundary of the trauma concept, as posited by the model, within the predictive processing framework (PPF). I integrate the PPF view with the theory of stress. In conclusion, I briefly discuss how the proposed model of trauma may guide clinical practice.

## Introduction

The construct of psychological trauma has been a subject of ongoing debate. In part, the difficulty to establish a consensus lies in the different facets of this concept that need to be reconciled and integrated. Trauma can refer to an event, experience, and symptoms, blurring the line between its cultural and medical meanings (Summerfield, 2001). Traditionally, there have been two trends in defining trauma. One is related to criterion A for acute and posttraumatic stress disorders (PTSDs) in the consecutive issues of the *Diagnostic and Statistical Manual of Mental Disorders, Fifth Edition* (*DSM-5*; American Psychiatric Association, 2013), which in *DSM-5* states “Exposure to actual or threatened death, serious injury, or sexual violence in one (or more) of the following ways…” (*DSM-5*, p. 271). This criterion establishes the severity threshold in search of a categorical boundary (Weathers and Keane, 2007). Such a boundary is crucial for operationalizing trauma for research and practice. McNally (2009) notes that “conceptual bracket creep” carries the danger of trivializing trauma by eroding its singularity.

Indeed, trauma is often conflated with adversity as exemplified by the definition of trauma as “any event that has had a lasting negative effect upon self and psyche” (Shapiro, 2017, p. 39). An implicit conflation of these categories is evident in their widespread interchangeable use in the literature. Thus, a Google Scholar search for “trauma *and* adversity” returned 4,490 “hits,” whereas “trauma *or* adversity,” only 632, most of which still did not differentiate between the two but used them as a compound concept. The need to distinguish between trauma and adversity has been demonstrated in research on childhood adversity and trauma (e.g., McCrory et al., 2010; McLaughlin, 2016). This body of research shows that different adverse childhood experiences lead to qualitatively different pathologies and that lumping them together under the category of trauma may be misleading. Theoretical, methodological, and practical reasons for differentiating trauma from adversity have been reviewed elsewhere (Krupnik, 2019).

Significant progress in understanding and treating PTSD, so that PTSD is now among the most responsive disorders to psychotherapy (Bradley et al., 2005), has likely been assisted by the narrow *DSM* definition of trauma. However, its limitations have also been noted. One concerns types I and II trauma and refers to the distinction between a singular traumatic event and cumulative trauma resulting from repeated (e.g., childhood) abuse (Terr, 1991). Another is that PTSD diagnostic criteria do not capture developmental trauma, and developmental trauma disorder has been suggested as a separate category to be included in the *DSM* (Van der Kolk, 2005). This calls for a more inclusive concept of trauma accommodating a wider scope of adverse experiences.

Whereas a more inclusive concept of trauma is likely to produce the very conceptual bracket creep that McNally warned against, some researchers have pursued this path developing a dimensional category of trauma. For example, the notion of PTSD as the highest degree of the normal stress response has been supported by a taxometric analysis of PTSD’s latent structure (Ruscio et al., 2002; Broman-Fulks et al., 2009). Likewise, researchers of posttraumatic growth propose a dimensional view of adversity, where PTSD is considered its highest degree (Seery et al., 2010).

The dimensional view has been taken to its logical end by completely erasing the boundary between trauma and adversity as exemplified by the notion of a continuum from “small trauma” to “big trauma” (Shapiro, 2017). The drive for hyperinclusivity of the trauma concept appears especially prevalent in the clinical realm, for example, “There is more to trauma than PTSD” (Shapiro, 2010, p. 11) or “It can perhaps be conjectured that unresolved trauma is responsible for a majority of the illnesses of modern mankind” (Levine, 2010, p. 184). A practical corollary to the hyperinclusive trauma concept is that trauma- and adversity-focused treatments become interchangeable. Indeed, cognitive–behavioral therapy (CBT) has been adapted to trauma treatment as trauma-focused CBT (Cohen et al., 2017) or CPT (Resick and Schnicke, 1993), and, on the other hand, eye movement desensitization and reprocessing, initially developed specifically for trauma, have since been adapted to a wide range of psychiatric and somatic conditions (Shapiro, 2009).

In a recent attempt to reconcile the dimensional and categorical views of trauma, a hybrid categorical–dimensional model of trauma was presented in the context of the general theory of stress (Krupnik, 2019), where trauma was defined as a particular kind of stress response, that is, the traumatic stress response (TSR) distinct from stress response to adversity, that is, the pathogenic stress response (PSR). Herein, I build upon this model to further specify the properties of TSR in the predictive processing framework (PPF).

## Trauma as a Stress Response

In the hybrid categorical–dimensional model, trauma is defined not as an event but the subjective experience of *stress*, that is, stress response. This experience is generated via afferent interoceptive pathways by sensing what happens in the body in response to external and internal disturbances or *stressors*. Interoceptive signals are integrated in the insular cortex, which represents the body’s internal states on a moment-by-moment basis and coordinates its homeostatic response to compensate for the disturbances and return to its “set points” (Craig, 2002, 2009). In case of failure to return to its homeostatic state under pressure from stressors, the organism undergoes allostasis (“stability through change”) (Sterling and Eyer, 1988; McEwen and Wingfield, 2003) to a new, suboptimal, homeostatic state that can lead to pathology. In such an outcome, the organism experiences allostatic overload, of which two types, types 1 and 2, have been identified (McEwen and Wingfield, 2003). Type 1 can be viewed as a situation where a disturbance overwhelms the organism’s coping resources, triggering an emergency response dramatically curtailing functions non-essential for immediate survival. Type 2 refers to a situation of a chronic stressor pressure that creates a drift away from the initial homeostatic state without triggering an emergency response.

**FIGURE 1:**
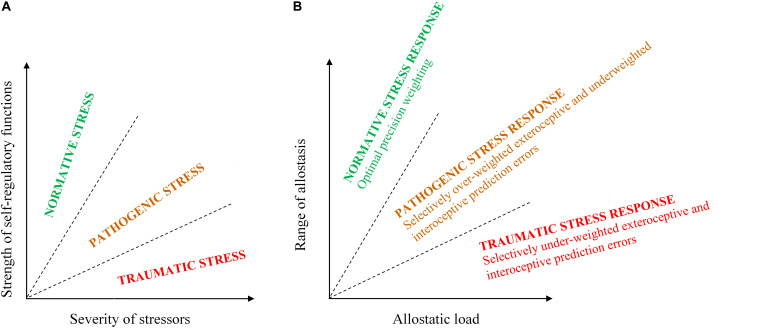
Hybrid categorical-dimensional model of stress **(A)** and stress response **(B)** continua (modified from Krupnik, 2019).

The hybrid model (Figure 1A) defines the stress continuum along two axes: severity of stressors and strength of self-regulatory functions; their ratio determines the nature of stress response, that is, its place on the continuum (Krupnik, 2019). The continuum is divided into three categories (Figure 1A): normative stress response (NSR), PSR, and TSR.

In NSR, the organism adapts to the stressors by returning to the optimal functional state, and no pathology ensues. Under allostatic overload, allostasis can proceed either through PSR, where type 2 overload results in a transition to a new, less adaptive, homeostatic state with self-regulatory functions relatively intact, or through TSR, where type 1 overload triggers an emergency response and a breakdown of self-regulatory functions^1^. A corresponding working definition of trauma was proposed (Krupnik, 2019, p. 259):

To be considered traumatic, a stress response to an event must meet a necessary condition that the event be outside of the person’s normative life experience, and a sufficient condition that the response include a breakdown of self-regulatory functions.

The main feature distinguishing TSR from PSR was identified as a breakdown of self-regulatory functions. Neurological markers of such breakdown were hypothesized to associate with a malfunction of the default mode network (Raichle, 2015) and clinical markers to largely overlap with PTSD symptoms (*DSM-5*). In the following sections, I explore the PSR/TSR difference in the PPF to further elucidate the functional basis of this distinction.

## Psychopathology in the Predictive Processing Framework

### The Predictive Brain

In a recent paradigm shift, brain is no longer viewed as a passive receiver and processor of information. Instead, it is thought of as a predictive coding machine operating by rules of Bayesian inferential statistics (Friston, 2010; Clark, 2013b). In short, brain continuously runs a generative model of the external (the world) and internal (the body) environment. The model predicts the incoming sensory stimuli, which reflect the states of the environment, by inferring their causes. It does so by embodying (through synaptic architecture and strength) the probability that the sensation S is caused by the event/circumstance E. For example, the brain of a mammalian infant embodies the high probability of a pleasurable sensation in the mouth following suckling on a nipple. The belief that S follows E is called a *prior* or prior belief (or prediction) and is thought of as a probability distribution function representing prior learning. Bayesian statistics establish a rule for updating the prior according to the likelihood that S does, indeed, follow E. The updated prior is called *posterior* (posterior belief). Thus, the model continuously updates itself through posterior learning. Prior beliefs are understood here in a broad sense, where they can reside at any level of information processing from unconscious expectancy to the declarative abstract thought. When sensory input does not match the prior, a *prediction error* (PE) is generated. Prediction error’s function is to update the brain’s generative model, which happens through resolving/suppressing the PE. This can happen in two ways through (a) *posterior learning* by adjusting the model’s prior to match the input or (b) *active inference* by adjusting the organism’s properties and/or behavior so that it controls the sensory input (“samples the environment”) in a way that matches the model’s prior. Through iterative cycles of perception–action, the brain directs the organism’s behavior to selectively seek and gate sensory information to fulfill its predictions. An example of active inference is mammalian infants deriving pleasure from suckling on an object, making it possible to “fool” them with a pacifier.

More recently, PPF has been integrated with the free-energy principle (FEP) (Friston et al., 2006; Friston, 2010). The FEP provides a causal link between the mechanics of PPF and the teleological evolutionary frame. Being a product of evolution, the meta-purpose of PP is adaptation to the environment by anticipating its demands and by maintaining an optimal structural organization to meet them. Accordingly, FEP postulates that an organism’s generative model is continuously increasing its accuracy by minimizing its variational free energy (informational entropy). Variational free energy is defined as the upper limit on surprisal or uncertainty about the brain’s sensory states. It is also related to structural entropy, providing a causal link to the second law of thermodynamics and, in turn, to the universal principle of self-organizing systems, which states that they self-organize by minimizing their entropy (Ashby, 1991). Thus, FEP has been suggested as a universal theory of brain, where all brain functions can ultimately be traced to minimizing its free energy (Friston, 2010).

In order to run an accurate generative model, the brain has to optimally weight its priors against PEs, which is known as *precision estimate or weighting* (Friston, 2009; Clark, 2013a). Both priors and PEs are defined as probability distribution functions, whose inverse variance is called *precision*; that is, high variance corresponds to low precision and *vice versa*. If the priors are too rigid (hyperprecise) they may be refractory to PE and fail to update according to the changing environment, rendering the generative model insensitive. On the other hand, too malleable priors (low precision) may lead to an unstable model, hypersensitive to environmental contingencies, and thus lacking in predictive power. Precision weighting is thought to be mediated by neuromodulatory control of the synaptic gain of PE units (Friston et al., 2014). Imbalance of precision weighting with overweighted or underweighted PE may result in false/inaccurate inference.

### Psychopathology as False Inference

Malfunction of predictive processing has been proposed as the universal etiology of psychopathology (Friston et al., 2014). Indeed, a growing number of psychiatric conditions have been conceptualized in PPF, including psychosis (Adams et al., 2013; Powers et al., 2017), depression (Barrett-Feldman et al., 2016; Badcock et al., 2017), anxiety disorders (Paulus et al., 2019), disorders of personality (Moutoussis et al., 2014), autism (Lawson et al., 2014), functional neurological disorders (Edwards et al., 2012), and attention-deficit/hyperactivity disorder (Dołȩga, 2018).

Malfunction of PP can happen in different interrelated domains: perceptive inference including exteroception and interoception and active inference including motor and visceromotor action, respectively. Suboptimal precision weighting leading to false inference has been identified as central to psychiatric etiology (Friston et al., 2014). For instance, false proprioceptive inference has been implicated in motor movement disorders (Edwards et al., 2012), whereas false exteroceptive inference, in psychosis (Adams et al., 2013; Powers et al., 2017). False interoceptive inference has mostly been implicated in mood/affective disorders (Paulus et al., 2019).

### Maladaptive Stress Response as False Inference

Psychopathology has also been conceptualized as inadequate stress response, which is widely known as the diathesis-stress model. The model posits that the combination of stress severity and vulnerability to stress determines whether stress results in resilience or pathology (Monroe and Simons, 1991). This presumes a continuum of stress response from resilience to pathology, similar to the one in Figure 1A. In allostasis terms, healthy versus pathologic stress response depends on how adaptively the organism negotiates allostatic load by adjusting its homeostatic states within the physiological range (Sterling, 2012). Self-regulation is believed to mediate such adaptability.

In the last decade, the theory of allostasis has been integrated into PPF. Survival in a changing environment is the ultimate goal of behavior; therefore, allostasis is the brain’s evolutionary purpose and primary function (Sterling, 2012). Accordingly, allostasis is understood as an organism’s ability to adjust its internal state to anticipated future challenges (e.g., increasing the blood pressure in preparation for standing up). Such anticipation is encoded by the brain’s generative model, specifically, its interoceptive inference. Thus, interoceptive inference has been proposed as the mechanism of allostasis (Barrett-Feldman and Simmons, 2015; Peters et al., 2017).

Interoceptive inference is underwritten, as discussed above, by the FEP stipulating that the brain’s ultimate function is to minimize the uncertainty about its sensory states (internal states in case of interoception). Then, the meaning of an adaptive stress response (successful allostasis) is an accurate prediction of internal states that optimally accommodate the future challenge.

## Predictive Processing Account of Trauma

Predictive processing framework has recently been applied to trauma (Wilkinson et al., 2017; Linson and Friston, 2019), where different manifestations of PTSD are regarded as a trauma-induced malfunction of the brain’s generative model. For example, dissociation is thought to result from the fragmentation of the generative model, where the model of the self is disconnected from the model of the traumatic event. Consequently, the self-model fails to be updated by the ascending interoceptive PEs (iPEs), making the trauma felt as happening not to oneself (Wilkinson et al., 2017). As a result, the negative affect is mitigated during a traumatic event, which may be adaptive as a peritraumatic response but becomes maladaptive long-term, making dissociation one of PTSD symptoms (*DSM-5*). On the other hand, the posttraumatic model of the world is believed to rely on hyperprecise predictions of threat that do not require sensory confirmation for triggering a metabolic “fight or flight” response (Linson and Friston, 2019). I think that the hypothesized insensitivity (underweighting) to PE is an essential property of TSR and will expound it further below.

### TSR as Type 1 Allostasis

Emergency response to an environmental challenge is one of the features of allostatic overload type 1 (McEwen and Wingfield, 2003). This emergency quality links type 1 allostasis to the necessary condition of TSR in the above definition of trauma; that is, “the event is outside of the person’s normative life experience.” Outside its normative range of experience, the organism’s generative model receives a sensory input that it predicts as highly improbable, which generates a hyperprecise PE. This is likely to trigger a dramatic increase in the model’s uncertainty/free energy. In order to compensate for it, just as dramatic a response may be required. The two available pathways, as mentioned above, are acting on the environment according to active inference or updating the priors through posterior learning, that is, adjusting the perceptive inference. In a traumatic event, action may not be available, which can render the event even more traumatizing. Then, the remaining option is an abrupt overhaul of the model’s network of priors, often leading to dysregulation within the model, for example, dissociation or psychosis (Wilkinson et al., 2017). Such dysregulation corresponds to “breakdown of self-regulatory functions” suggested as the sufficient condition and a defining feature of TSR (Krupnik, 2019).

Type 2 overload, on the contrary, is associated with a lower intensity chronic challenge to the generative model (McEwen and Wingfield, 2003), which would be associated with lower precision PEs that may not be out of range of its priors. This may present an opportunity for a gradual response combining a compensatory active inference with a measured adjustment of perceptive inference. Such a response may still prove suboptimal and entail pathology (PSR), because the allostatic load may still “lock” the organism in a limited/forced range of stress response (Juster et al., 2010). I suggest that the lack of pathology differentiates NSR from both PSR and TSR, whereas the nature of PP malfunction differentiates PSR from TSR (Figure 1). Below, I specify the kind of malfunction I hypothesize to differentiate TSR from PSR^2^.

### TSR as Dysregulation of Precision Weighting

**FIGURE 2:**
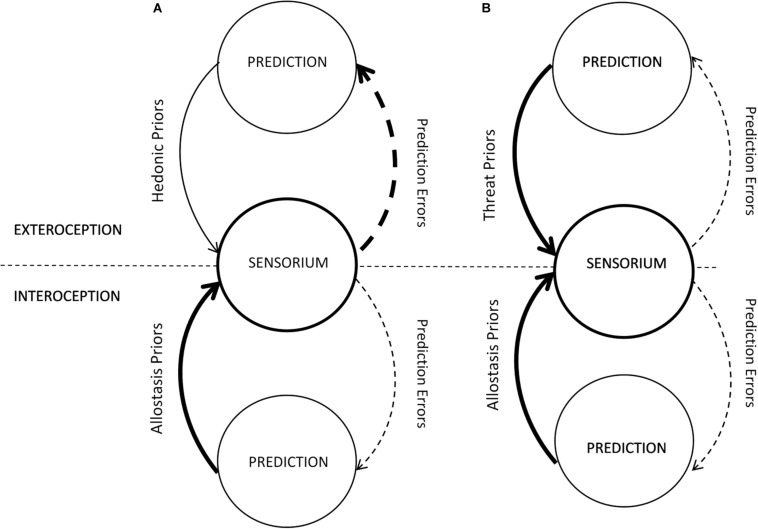
Imbalanced precision-weighting in pathogenic/depressive **(A)** and traumatic **(B)** stress response (modified from Krupnik, 2020). Arrow thickness denotes the relative precision.

I hypothesize that in TSR the generative model of the world undergoes a drastic recalibration of its predictions (exteroceptive priors). As a result, the model generates a set of hyperprecise predictions of the world’s dangerousness to accommodate the traumatic experience in a way that suppresses the PE, thus minimizing the model’s free energy (Figure 2B). Such recalibration results in a biased exteroceptive inference about the world as inherently threatening, which mindset is a hallmark manifestation of PTSD (*DSM-5*) including symptoms of hypervigilance and unrealistic negative beliefs about the world. Hyperprecise “threat” priors will result in relatively underweighted exteroceptive PEs (ePE). I suggest that, in PPF, precision weighting functions as a mechanism of allostasis, where the ratio between a prior and the PE precisions determines the current homeostatic set point for the prior, that is, its mean and variance. Under allostatic load, these parameters change in a way that minimizes the model’s free energy.

Trauma-induced exteroceptive “blindness” is expected to be trauma-specific; however, it may generalize to unrelated or loosely related sensory information. For example, PTSD patients show hyperactivation of the amygdala in response to both trauma-specific and neutral images. Moreover, this hyperreactivity is observed even without recognition of presented stimuli, indicating that it is likely due to the top-down, that is, prediction-driven, information processing (Hendler et al., 2003).

Peritraumatic reaction to type 1 allostatic overload is associated with an extreme visceromotor response commonly known as “fight, flight, or freeze” (Barlow, 2004), which can be regarded as a visceromotor active inference about a life-threatening situation (Hutchinson and Barrett, 2019). If so, such inference is expected to return to its normative range once the threat is over. However, if the mind is under control of hyperprecise exteroceptive “threat” priors, the threat never ceases (Wilkinson et al., 2017). Consequently, to minimize uncertainty, interoceptive inference needs to match the exteroceptive one by, too, becoming hyperprecise in predicting an emergency stress response (Figure 2B). This would lead to selective underweighting of iPE and, consequently, “lock” the mind in a self-perpetuating cycle of TSR, where hyperprecise exteroceptive and interoceptive priors feed into each other. Indeed, such a hallmark PTSD symptom as hypervigilance (*DSM-5*) can be seen as both an active inference (hypervigilant behaviors) “fulfilling” the hyperprecise exteroceptive “threat” priors and interoceptive active inference (visceromotor manifestations) “fulfilling” the hyperprecise interoceptive predictions of physiological emergency response (Figure 2B).

I suggest that the described dysregulation of precision weighting in TSR differentiates it from PSR (Figure 2) and, consequently, differentiates trauma from adversity. PSR results from allostatic overload type 2, where the organism faces no acute survival/reproductive threat but is under environmental pressure that may drive it into a suboptimal homeostatic range. This does not necessitate an abrupt recalibration of the priors into hyperprecise “threat” predictions. Instead, the generative model may decrease the priors’ precision to better accommodate the higher range of error and minimize the uncertainty in that way. This will result in a *relative* increase of ePE’s precision and their overweighting in the service of allostasis (Figure 2A). As in TSR, I expect such overweighting to be stressor-specific to a degree but allow for its generalization. For example, in generalized anxiety disorder, people react with anxiety to a large variety of external and internal stimuli with a higher share of miscellaneous items compared to controls (Roemer et al., 1997).

Overweighting of ePE presents a challenge for the interoceptive inference. In an inherently uncertain world (high-precision ePE), it is difficult to predict an appropriate visceromotor response. This challenge was aptly expressed in application to depression; “major depression occurs when the brain is certain that it will encounter an uncertain environment; that is, the world is inherently volatile, capricious, unpredictable, and uncontrollable” (Clark et al., 2018, p. 2278). Two opposite strategies can be envisioned for interoceptive inference to “keep up.” One is to “relax” the precision of its priors and accommodate/overweight the iPE. This would lead to the organism’s overreaction to its “noisy” internal sensation, as was previously hypothesized (Barrett-Feldman et al., 2016; Clark et al., 2018). The other would be to increase interoceptive priors’ precision, making the generative model relatively insensitive to internal sensations (and iPE) and thus ever ready for allostatic overload as in TSR. The latter strategy was also recently hypothesized (Krupnik, 2020). Indeed, chronic hyperarousal has been noted in anxiety and depression (Greaves-Lord et al., 2007). Hence, interoceptive inference can compensate for the overweighting of ePE by underweighting iPE (Figure 2A). Accordingly, the proposed model of the stress continuum (Krupnik, 2019) can be developed into a model of *stress response continuum* (Figure 1B). Because the stress continuum model defines stress as the organism’s experience of environmental challenges (stressors), it follows that a stress response continuum model defines stress response as the organism’s (and its brain’s) response to that experience, or allostasis. Then the axes determining the stress continuum (Figure 1A) can be transmuted to frame the stress response continuum. The strength of self-regulatory functions in allostasis terms denotes the range of adaptive physiological reactions to stress or, in PP interoceptive terms, the predicted range of allostasis; hence, this axis can be transmuted into “range of allostasis.” Likewise, in allostasis terms, the severity of stressors translates into allostatic load, which becomes the other axis of the continuum (Figure 1B).

The above model of stress response may also be applicable to its developmental dynamics. This aspect, although important and far-reaching, is beyond the scope of this article and will be addressed only cursory. Stress response is both constrained and biased by innate and genetic factors, which may determine the organism’s allostatic range. Genetic differences in the stress responsivity have been observed since long ago in strains of rats (Sternberg et al., 1992). Stress response is also learned from the perinatal stage onward (for review, see Novais et al., 2017). More recent research shows how different kinds of early childhood adversity shape reactions to stress and psychopathology later in life (Machlin et al., 2019). Therefore, an individual style of the stress response may be considered an endophenotype. Here, I want to highlight self-efficacy as a potentially central element of such an endophenotype. The crucial role of allostatic self-efficacy in depression and fatigue has been hypothesized before (Stephan et al., 2016). Self-efficacy has also been proposed as a meta-prior playing a pivotal role in precision-weighting regulation during stress response (Krupnik, 2020).

In order to elaborate the described model in greater clinical detail, below, I compare TSR to PSR, using examples of their prototypical clinical conditions, PTSD and depression, respectively. A detailed description of the PSR model of depression can be found elsewhere (Krupnik, 2020).

## PTSD and Depression as Different Kinds of Stress Response

Despite their high comorbidity (Bleich et al., 1997), depression and PTSD are clinically distinct. One of the hallmarks of PTSD is avoidance (*DSM-5*), where patients avoid reminders, thoughts, and feelings associated with the traumatic event. Depressed people, on the contrary, tend to engage in rumination, that is, prolonged reminiscing and brooding about their past and present misfortune (Nolen-Hoeksema et al., 2008). This contrast is further underscored by the difference between neurological signatures of depressive rumination and recall of traumatic memories. The latter is associated with deactivation of the medial prefrontal cortex in PTSD patients (Lanius et al., 2002), whereas the former shows increased activity in this area (Nolen-Hoeksema et al., 2008).

The significance of these differences for the stress response model is several-fold. The medial prefrontal cortex is part of the default mode network, which plays a central role in self-regulation (Raichle, 2015). Its inhibition during the processing of disturbing information in PTSD is consistent with the malfunction of self-regulation proposed as the main feature of TSR in the definition of trauma (Krupnik, 2019). Breakdown of self-regulation, including peritraumatic and posttraumatic dissociation, has been long associated with trauma and PTSD (Van der Kolk and Van der Hart, 1989). Neither impaired self-regulation nor dissociation has been noted as core features of depression; moreover, the adaptive value of depressive stress response including rumination has been highlighted by some researchers (Nesse, 2000; Gilbert, 2006; Andrews and Thompson, 2009).

Linson and Friston (2019) suggest that a traumatized generative model is characterized by decoupling of top-down priors from the bottom-up sensory information processing, which makes the organism react to the sensory cues according to the “threat” priors. This decoupling is seen as the mechanism of impaired reality testing observed in such PTSD symptoms as hypervigilance and increased startle response (to auditory stimuli, in particular) and implies selective underweighting of ePEs. These reactions along with experiential avoidance can be interpreted as an aberrant active inference “fulfilling” the hyperprecise threat priors (Figure 2B).

On the interoceptive side, PTSD is characterized by chronic hyperarousal (*DSM-5*), which in PPF can be regarded as active interoceptive inference meant to minimize the uncertainty of a generative model that overpredicts threat. Selective underweighting of iPE helps sustain the hyperprecise “allostasis” priors. Thus, PTSD generative model is defined by a combination of “runaway” exteroceptive “threat” and interoceptive “allostasis” priors (Figure 2B), which makes it prone to recreating the virtual traumatic experience when triggered. This may explain “re-experiencing,” the most specific PTSD symptom (*DSM-5*). Noteworthy, such recreating often happens in night dreams, when there is little to no sensory input and, consequently, no ePE.

There are several accounts of depression in PPF (Chekroud, 2015; Barrett-Feldman et al., 2016; Badcock et al., 2017; Fabry, 2019; Kube et al., 2019). They point to a commonality between depressive and PTSD generative models. Both have been characterized by hyperprecise interoceptive allostasis priors and underweighted PE (Figure 2), which maintain the organism in a state of allostatic overload in anticipation of an inimical/dangerous environment. Such a state has been dubbed a “locked-in” brain (Barrett-Feldman et al., 2016) or selective *interoceptive blindness* (Krupnik, 2020). In depression, it manifests in increased anxiety and hyperarousal (e.g., insomnia) instead of hypervigilance. There have also been suggestions that depression is associated with overweighted iPE (Clark et al., 2018), and indeed hypoarousal and lethargy are also featured in depression (Greaves-Lord et al., 2007). The dynamic model of depression resolves this apparent contradiction by taking into account different phases (with different manifestations) of the depressive stress response (Krupnik, 2014, 2020).

Where depressive and PTSD generative models differ is exteroceptive inference (Figure 2). Without the need for an abrupt recalibration of exteroceptive priors (as in trauma), the organism can use the alternative strategy of *decreasing* the precision of its exteroceptive priors to better accommodate the depressogenic ePE and thus minimize the model’s uncertainty. The ensuing overweighting of ePE may explain such clinical manifestations of depression as rumination about environmental challenges as opposed to avoidance, psychomotor retardation, and indecisiveness as opposed to increased startle response (*DSM-5*).

In accord with the highlighted differences between TSR and PRS generative models (Figure 2), I suggest to specify the stress continuum (Figure 1B) and update the earlier (Krupnik, 2019) definition of trauma in the following way:

To be considered traumatic, a stress response to an event must meet the necessary condition that the event be outside of the person’s normative life experience, causing an abrupt recalibration of exteroceptive priors, and the sufficient condition that the response include a breakdown of self-regulatory functions manifested in a malfunction of predictive processing with selectively underweighted precision of exteroceptive and iPEs.

## Empirical Evidence

Whereas the described model of TSR (Figures 1, 2) is speculative and has not been directly tested, much of empirical knowledge about trauma appears consistent with it. The evidence that trauma and PTSD, in particular, are associated with false inference, where hyperprecise threat priors skew the perception (sometimes to the point of flashback and hallucination), has been reviewed before (Wilkinson et al., 2017). Likewise, false interoceptive inference in PTSD has been extensively discussed (Linson and Friston, 2019). Here, I want to highlight perhaps the most direct indications of impaired predictive processing in trauma. Two related studies explored the encoding of PE in trauma (Lenow et al., 2014; Ross et al., 2018). In a reinforcement learning task, the authors demonstrated a decreased PE encoding in the medial PFC/ventral striatum network and anterior insula in people with PTSD (Ross et al., 2018). Low anterior insula PE encoding in traumatized individuals was also observed in a trust violation paradigm (Lenow et al., 2014). The medial PFC/ventral striatum network is responsible for assigning value to external information (Rushworth and Behrens, 2008), whereas the anterior insula is considered the hub of interoception (Craig, 2002). These data support my suggestion that a traumatized generative model is characterized by selective underweighting of ePE and iPE (Figure 2B).

Depression, too, has been associated with low interoceptive sensory sensitivity, that is, underweighted iPE. Phenomenologically, it is manifested in the emotional flatness of depressed people (Rottenberg et al., 2005). Depressed patients show decreased activity in the insula during an interoceptive task (Avery et al., 2014). The impaired interoceptive accuracy in depression has been documented in a recent comprehensive review (Eggart et al., 2019). The cumulative findings show a correlation between low affectivity and low interoceptive accuracy. Interestingly, the largest interoceptive deficit is noted in moderate depression, whereas it normalizes in severe one. This seemingly paradoxical finding fits the dynamic PPF model of depression (Krupnik, 2020)^3^.

The central claim of my model is that, in contrast to underweighted ePEs in trauma, depression is associated with their selective overweighting. According to Badcock et al. (2017), it may result from suspended sensory attenuation due to impaired neuromodulation by dopamine and serotonin. The converging evidence of impaired ePE processing has recently been systemically reviewed (Kube et al., 2019). Specifically, depressed patients show increased loss-related PE encoding in the ventral striatum (Ubl et al., 2015). In contrast to PTSD, depressed patients appear to have an intact reward PE encoding in the ventral striatum (Rutledge et al., 2017). Applying the work on PE dynamics (Kiverstein et al., 2019) to depression, Fabry (2019) explores the hypothesis that the depressive generative model is caught in a cycle of overestimating the rate of PE minimization, leading to accumulation of a “larger-than-expected” (i.e., overweighted) PE.

In aggregate, the existing evidence points to the qualitative difference between neural dynamics in PTSD and depression. The suggested model (Figure 2) interprets this difference in PE processing terms, contrasting traumatic with pathogenic (Figure 1) stress response. More research is needed to further clarify the difference in PPF terms between PTSD and depression, specifically by direct comparison of respective patients in the same experimental paradigm.

## Discussion

Perhaps the most significant aspect of the suggested model of stress continuum (Figure 1) is its practical application. Specifically, the different nature of TSR versus PSR implies that different therapeutic approaches may be efficacious in either case. This goes against the late trend toward universal psychotherapies. For example, CBT has been used and found efficacious for a plethora of mental conditions, and its various modifications have cropped up including trauma-focused CBT (Cohen et al., 2017). The opposite tendency, that is, toward specificity of psychosocial interventions for their target, also has its proponents (e.g., Parker et al., 2003).

The proposed model implies different therapeutic strategies for TSR and PSR. All efficacious therapies for PTSD have exposure at their core. This appears a straightforward approach because it allows for reshaping the patient’s perceptual and active inference (including interoceptive) response by changing the external and internal context of traumatic imagery, as well as behavioral reaction to it. On the other hand, using exposure for the treatment of depression may not provide significant benefits because, unlike PTSD patients, depressed people do not avoid the negative material but self-expose to it through rumination (Nolen-Hoeksema et al., 2008). Accordingly, it was proposed that instead of the negative material the dynamics of depressive response may be a better target for intervention (Krupnik, 2014), and a corresponding modification of a trauma-focused therapy has been developed for depression (Krupnik, 2018).

## Author Contributions

The author confirms being the sole contributor of this work and has approved it for publication.

## Conflict of Interest

The author declares that the research was conducted in the absence of any commercial or financial relationships that could be construed as a potential conflict of interest.

## Footnotes

1 Of note, the proposed categories of stress response are similar to McEwen’s model which distinguishes “good,” “tolerable,” and “toxic” stress (McEwen and Gianaros, 2011), although the model is based on a different theoretical premise.

2 I want to emphasize that the proposed malfunction need not be limited to TSR but may be found in other pathologies; it only becomes a trauma attribute when caused by a stressor outside the organism’s normative range of experience.

3 The dynamic model also accommodates the related paradox known as “the dark room paradox” as it applies to depression (Fabry, 2019).
